# Teachers' reported beliefs about giftedness among twice exceptional and culturally, linguistically, and economically diverse populations

**DOI:** 10.3389/fpsyg.2022.953059

**Published:** 2022-08-08

**Authors:** Rachael A. Cody, Gregory T. Boldt, Elizabeth J. Canavan, E. Jean Gubbins, Stacy M. Hayden, Aarti P. Bellara, Kelly L. Kearney

**Affiliations:** ^1^Neag School of Education, University of Connecticut, Storrs, CT, United States; ^2^The Ohio State University, Columbus, OH, United States

**Keywords:** twice exceptional, gifted and talented students, teacher beliefs, linguistically and culturally diverse student populations, economically diverse populations, identification, pedagogical programming

## Abstract

The purpose of this research study was to assess the impact of professional learning on teachers' reported beliefs about students identified as twice exceptional (2e) and students from culturally, economically, and linguistically diverse (CLED) populations, using a semi-randomized experimental design intervention. Teachers in the experimental condition participated in professional learning opportunities featuring curriculum materials, lessons, and activities highlighting support for students identified as 2e or from CLED populations. Teachers in the control condition received no intervention. Across 16 United States' schools, 53 grade 3 classroom teachers were selected to complete two sets of pre-intervention and post-intervention surveys assessing their reported beliefs about students identified as 2e or from CLED populations. The results indicated that all teachers consistently reported accurate and positive beliefs about the characteristics and needs of these populations, both prior to and after participation in relevant professional learning opportunities. Although analyses revealed main effects of condition and time for certain scales, the reported interaction terms suggested that the professional learning opportunities did not specifically increase questionnaire scores for teachers in the experimental condition. The implications of these findings regarding professional learning and efforts to improve equity in gifted and talented education are discussed.

## Introduction

Researchers and practitioners alike are becoming increasingly aware of the underrepresentation that plagues gifted and talented programs, especially concerning students identified as twice exceptional (2e) and students from culturally, linguistically, and economically diverse (CLED) populations, where underrepresentation may more significantly influence the extent to which these students are underserved in their academic environments (Siegle and McCoach, 2010; Hamilton et al., 2018). Although the terms 2e and CLED describe distinct and varied student populations, both groups remain chronically underidentified as gifted and underrepresented in gifted programs, and professional learning opportunities are needed to address variations in teachers' understanding of these populations (Siegle et al., 2016). Researchers in the field of gifted education (Anthony et al., 2009) have described traditional characteristics of gifted students that include high verbal abilities, early reading abilities, keen powers of observation, strong critical thinking skills, problem-solving skills, decision-making skills, sensitivity, intense concentration, questioning attitudes, creative skills, tendencies to take risks, a highly developed sense of humor, and a sense of independence. The expression of these characteristics may not, however, be universal, which has prompted researchers to investigate the ways in which students of differing populations may exhibit these high potential behaviors. The purpose of this article is to examine teachers' beliefs about students who have been historically underrepresented in gifted and talented programs. More specifically, this study examined teachers' reported beliefs about students identified as 2e, and students from CLED populations in grade 3 general education classrooms.

Gifted characteristics are present in students from 2e and CLED populations, but they may be demonstrated in behaviors that teachers do not typically associate with giftedness. Teachers may value achievement over aptitude and conformity to classroom norms when considering students “high potential, despite researchers” understanding that these characteristics do not always indicate giftedness (Al-Hroub and Whitebread, 2008). In a study that examined Arabic and mathematics teachers' nominations of dual exceptional children, Al-Hroub and Whitebread found that teachers often focused on student characteristics such as school performance, achievement, contribution in the classroom, and interest in studying. Such generalizations are often inaccurate due to the diversity and intersectionality of 2e and CLED populations, and it is more pertinent to examine behaviors on an individual basis and in specific subpopulations. For example, students who are English learners (ELs) may possess excellent critical thinking skills that they only manifest when speaking or writing in their native languages. Teachers may not be able to recognize students' critical thinking abilities if they hold a deficit-based view of their students that fails to address individual needs. However, when students engage with high-quality curriculum and concrete materials that are inclusive of cultures and languages, ELs may be better able to illustrate their developing thought processes and cognitive strengths (Siegle et al., 2016). In such cases, teachers can learn to recognize students' gifted potential prior to students' mastery of the English language (Siegle, 2020). Likewise, African American students may engage their problem-solving, critical thinking, concentration, and creative skills more often when engaging with others, as opposed to when working individually. African American culture and behavior are centered around pillars such as harmony, communalism, movement, and expressive individualism (Harmon, 2001). These pillars are oppositional toward traditional classroom expectations that center around perfection, competition, passivity, and conformity (Okun, 2021).

Similar issues arise for students from low socioeconomic backgrounds (consider situations in which a student provides extremely curious comments in class that seem off-task to the teacher). Teachers may not understand the cultural differences that coincide with low socioeconomic backgrounds and may use this misunderstanding to form their perceptions of students' abilities. Olszewski-Kubilius and Clarenbach (2012) echoed this sentiment, stating that inaccurate perceptions are one of the most significant educational barriers facing high ability learners from low socioeconomic backgrounds. Therefore, teachers and gifted program directors should recognize the cultural differences that lead to the underrepresentation of students from CLED backgrounds in gifted programs and implement curriculum/instructional strategies to increase program participation for these students (Briggs et al., 2008).

Specifically, 2e refers to “students who are identified as gifted and talented and are also diagnosed with one or more of the special education categories that are defined by the Individuals with Disabilities Education Act, except in cases where students demonstrate cognitive disabilities” (Reis et al., 2014, p. 219). Teachers may find that students identified as 2e exhibit gifted behaviors that align with teachers' positive perceptions of giftedness, such as creativity, critical thinking, curiosity, and problem-solving, but become frustrated with the academic challenges and behavioral difficulties that often permeate the 2e experience (Nielsen and Higgins, 2005). The National Education Association (2006) recognizes many possibilities that arise when discussing the instruction of students identified as 2e. students' academic gifts may mask disabilities, their disabilities may mask gifts, or masks and disabilities may work simultaneously to create the illusion of an average experience (Baum et al., 2017). In truth, students identified as 2e [specifically those who are diagnosed with a learning disability (LD)] may demonstrate cognitive and achievement characteristics marked by greater variance and more pronounced discrepancies between strengths and deficits than seen in individuals who are either gifted without a disability or of average ability (Maddocks, 2020). This can be seen in a nationally representative standardization sample for the co-normed Woodcock-Johnson IV Tests of Cognitive Abilities and Achievement. Results indicated that students identified as 2e with a LD exhibited higher achievement and cognitive scores than their average ability peers in areas related to verbal abilities and fluid reasoning, and these scores were similar to or slightly lower than those of their gifted peers who did not have a learning disability (Maddocks, 2020). However, students identified as 2e-LD showed abilities that paralleled average-ability groups in areas related to short-term working memory, auditory processing, and long-term retrieval. Additionally, students identified as 2e-LD demonstrated lower scores than average-ability groups in all achievement and cognitive tests that included a processing speed component. This wide range of abilities illustrates the broad range of services that students identified as 2e may require, as well as their need for enhanced teacher understanding (Baum et al., 2017). Sadly, teachers often direct their attention to students' deficits instead, overlooking the students' academic strengths.

Students identified as 2e often manifest “problem behaviors” such as laziness, willfulness, and a lack of attention that diminish over time when their instructional needs are met (Willis, 2012). Additionally, Reis et al. (2021) noted that students identified as 2e may face common challenges, such as difficulties engaging in social interaction, attention deficits, emotional maladjustment, and excessive focus on specific interests. These behaviors may not always be pleasing to the teacher and could adversely influence teachers' perceptions of students identified as 2e. Teachers are often provided gifted “trait lists” that omit potentially negative or dysfunctional behaviors that students who are 2e may exhibit, which can lead to a lack of support within the general education classroom (Al-Hroub and Whitebread, 2008). Additionally, Missett et al. (2016) noted one teacher in their study prevented a student identified as 2e from participating in group activities based on a deficit-based expectation, despite a lack of supporting evidence that this student was struggling. Missett et al. emphasized participating teachers focused on behavioral and academic deficits of students diagnosed as 2e rather than their strengths. This notion can be applied to beliefs about students from other underserved populations, as well. Mun et al. (2020) suggested that teachers and administrators hold implicit beliefs about students who are ELs that could negatively influence these students' nominations to gifted and talented programs. Because the nomination process neglects to cover the many possible facets of giftedness that may constitute the experiences of many students with gifts and talents (Leroux and Levitt-Perlman, 2000; Hamilton et al., 2018), it is likely that teachers' implicit beliefs disproportionately influence students from underserved populations. Ezzani et al. (2021) explained that the widespread underrepresentation for specific student populations denotes a systemic failure to understand how many students in underserved populations exhibit gifted characteristics. To better recognize expressions of giftedness in these populations, teachers must not confuse issues involving student access to gifted programming with student potential.

There are many barriers that may perpetuate the underrepresentation of students from historically underserved communities in gifted programs, such as outdated policies and procedures, inequitable intelligence and/or achievement tests, and disproportionate teacher referral rates (Ford, 2010; Siegle et al., 2016). According to Al-Hroub (2013), teachers often incorrectly nominate students, resulting in the provision of inappropriate educational services. Additionally, Mun et al. (2020) confirmed that teacher beliefs and biases about specific student populations negatively influence teacher nominations for gifted programming. To enhance opportunities for students to participate in gifted programs and to develop their gifted potential, Siegle et al. (2016) recommended that teachers change their attitudes to reflect positive perceptions about students in diverse populations and the gifted potential they may demonstrate. Poor educational outcomes for students from CLED populations have been frequently attributed to cultural mismatch between students and their teachers and many students identified as 2e believe themselves to be more frequent recipients of teacher bullying and yelling, recalling many instances of negativity and pain in their early educational experiences (Reis et al., 2000; Ford and Trotman, 2001; Ronksley-Pavia and Townend, 2017).

Acknowledging the influence of teachers' beliefs on students' educational experiences, research involving students identified as 2e and students from CLED populations has often focused on interventions targeting negative teacher perceptions of these populations. Although relatively sparse, these studies have yielded promising results. For example, Harradine et al. (2014) found that teachers claimed the Teacher's Observation of Potential in Students tool helped them notice Students of Color, increase positive teacher perceptions of them, and effectively respond to their needs. Other researchers, such as Nielsen and Higgins (2005), have highlighted the need for teachers to demonstrate compassion toward students identified as 2e, which may help to create respectful environments conducive to academic growth. Researchers who focus their efforts on teacher perceptions involving students identified as 2e and students from CLED populations may serve an important role in combating underrepresentation for underserved student populations, provided that their studies document the reliability and construct validity in the measurement of teacher perceptions. Without such validity, attempts to reverse underrepresentation may prove to be futile. Even with sound instruments, an exclusive focus on the improving teacher beliefs may not be sufficient to enable students to overcome the barriers in their academic environments. Rather, research coupling teacher beliefs with teacher practices may be necessary to accurately identify and develop the talents of students with gifts and talents from diverse backgrounds.

The theoretical framework of this study was social constructivism, which, “influenced by Vygotsky's (1978) work, suggests that knowledge is first constructed in a social context and is then internalized and used by individuals” (Amineh and Asl, 2015, p. 10). Social constructivism recognizes that individuals interact with each other and agree on interpretations of knowledge through both discourse and non-verbal communication (Bozkurt, 2017). Educational researchers who espouse this theory (Liu and Chen, 2010) have claimed that teachers socially construct their understanding of students and their potential, internalize this understanding, and use that understanding to inform their decisions about instructional practices. Specifically, Liu and Chen argued that educational research guided by social constructivism may be able to determine whether teacher beliefs are able to influence student learning. In this study, the social constructivist theory was used to shape our understanding of teachers' identification and instruction of gifted students from historically underserved populations.

The purpose of this study was to examine teachers' reported beliefs about students identified as 2e and students from CLED populations in grade 3 general education classrooms. Guided by the social constructivist theory, the researchers used instruments that surveyed teachers' existing beliefs about gifted characteristics in students from these populations. The specific questions guiding this study were as follows: (a) Do teachers' reported beliefs about giftedness in students identified as 2e improve after participation in relevant professional learning opportunities? (b) Do teachers' reported beliefs about giftedness in students from CLED populations improve after participation in relevant professional learning opportunities?

## Materials and methods

### Participants

This study included 53 grade 3 general education teachers sampled from 16 schools in the United States. Because the study was part of a multi-year grant during the COVID-19 pandemic, recruitment and randomization procedures varied slightly between schools. Three schools were recruited during Year 2 of the grant while the remaining 13 were recruited during the Year 3 of the grant, which is when the present study took place.

Of the 53 participating teachers, five experimental condition teachers and four control condition teachers continued their participation from Year 2, three new teachers joined in schools that began participating in Year 2, one teacher assigned to the control condition in Year 2 was reassigned to the experimental condition in Year 3, and the remaining 40 teachers were recruited within newly participating schools. Condition assignment was randomized in 10 participating schools and partially randomized in one school. To accommodate individual school need, random assignment was not possible across all the schools; thus, the study was not a true experimental study. Several school administrators did not agree to randomization conditions. Within other schools, it was not feasible to deliver intervention-specific professional learning (e.g., in the contexts of continuing schools where professional learning sessions had taken place the previous year). Altogether, 28 teachers were assigned to the experimental condition and 25 teachers were assigned to the control condition. Participants were aware of their condition assignment.

### Procedure and design

Teachers (*n* = 28) in the experimental condition implemented the Thinking Like Mathematicians (TLM): Challenging All Grade 3 Students study within their classrooms. The math unit, entitled *If Aliens Taught Algebra: Multiplication and Division Would be Out of this World!* (Cole et al., 2019) was purposefully designed based on two conceptual frameworks. The first conceptual framework ensured the math unit served as job-embedded professional learning and provided exemplars of pre-differentiated, enriched, and challenging math content to promote talent development. To accomplish this goal, the idea of creating educative curriculum material and promoting pedagogical content knowledge was essential. This led to a reliance on the work by Davis and Krajcik (2005) who emphasize how educative curriculum helps teachers think about students' responses to instructional activities, supports teachers' learning about the content, highlights the curriculum developers' pedagogical judgments, and fosters teachers' “pedagogical design capacity” (p. 5) to use personal and curricular resources to promote instructional goals.

With job-embedded professional learning integrated throughout the math unit and supported by 2 days of onsite activities, experimental teachers also re-visited three curriculum models associated with gifted education and talent development, which served as the second conceptual framework. These models included Differentiation of Instruction Model (Tomlinson, 2001), which promotes rich, engaging curriculum responsive to the learning needs of diverse learners. This perspective is aligned with the Depth and Complexity Model (Kaplan, 2005), which highlights the importance of considering the depth, complexity, abstractness, and acceleration of curriculum that incorporates advanced thinking skills, product development, and resources. The third model was the Schoolwide Enrichment Model (Renzulli and Reis, 2014), which encourages students to think, do, and act like practicing professionals or disciplinarians even at a younger age. The math unit highlighted the importance of students thinking like mathematicians as they developed their understanding and expertise with algebraic thinking, multiplication, and division.

The math unit comprised 16 lessons related to algebraic thinking, multiplication, and division. Within these lessons, 11 were pre-differentiated and enriched for the grade 3 general education classroom. Teachers were provided a pacing chart for both sequential implementation (i.e., 5 days a week for 5 weeks) and 4-month implementation (i.e., approximately one lesson a week). Teachers in the experimental condition were expected to follow one of the provided pacing charts for their implementation of the unit. They participated in 2 days of professional learning, totaling 14 h. These sessions explored mathematics lessons, activities, and curricular materials designed to meet the needs of high-potential students from diverse populations. The professional learning sessions encouraged participants to engage with and learn about the pre-differentiated and enriched TLM unit and gifted and talented identification and services, specifically for students identified as 2e and students from CLED populations. Teachers also examined student work samples and discussed identifying the talents and gifts of their students.

### Measures

The TLM research team developed a survey titled Teachers' Beliefs About Twice-Exceptional Students. After several rounds of item writing and consulting with external experts on 2e, a final survey that included 22 items across a 6-point Likert scale was developed. These 22 items measure two scales. The first scale measured teachers' beliefs about the characteristics and behaviors of students identified as 2e with 12 items, providing a summed score ranging from 12 to 72. The remaining 10 items comprised the second scale, teachers' beliefs about pedagogical programming for students identified as 2e, providing a summed score ranging from 10 to 60. As the survey items aligned with existing research on the characteristics and behaviors of students identified as 2e (e.g., “Twice-exceptional students demonstrate uneven academic skills”) and pedagogical programming strategies that best support these students (e.g., “Twice-exceptional students will benefit from gifted programming focused on their talents”), higher scores reflect more accurate beliefs about the characteristics and needs of this population. A final survey item prompted teachers to indicate whether they have instructed students identified as 2e. The survey scores demonstrated appropriate reliability, as measured by Cronbach's (1951) alpha, for both the student characteristics (pre α = 0.78; post α = 0.82) and pedagogical programming scales (pre α = 0.85; post α = 0.93).

The Teachers' Beliefs About Culturally, Linguistically, and Economically Diverse Gifted Students Survey, developed and psychometrically investigated by de Wet (2006), includes 21 items rated on a 5-point Likert scale representing three distinct factors: benefits of including students from CLED populations in gifted programs (nine items), universality of abilities (six items), and assessment of abilities (six items). The research team chose to exclude items from the scale that were beyond the scope of the study and added one item pertaining to the benefits of including students from CLED populations in gifted programs scale: “The inclusion of CLED students in gifted programs will enhance the multicultural nature of students' learning experience.” With these modifications, the final survey in this study measured teachers' perceptions regarding the benefits of including students from CLED populations in gifted programs (10 items) and the universality of abilities (six items).

### Statistical analysis

Descriptive statistics for individual survey items and scales were calculated to examine teachers' reported beliefs about students identified as 2e and students from CLED populations. Separate two-way, mixed ANOVAs investigated 2e and CLED survey scale differences, with administration time representing a within-subjects factor and condition representing a between-subjects factor.

## Results

### Teachers' beliefs about 2e students survey

#### Descriptive statistics

Collapsed across time periods, teachers reported accurate beliefs about both the characteristics and behaviors of students identified as 2e (*M* = 55.82, *SD* = 6.85) and pedagogical programming for students identified as 2e (*M* = 52.32, *SD* = 6.18), considering the maximum scale scores of 72 and 60, respectively. The scales were moderately correlated (*r* = 0.48, *p* < 0.001). In the initial survey, 45% of teachers (*n* = 24) reported having worked with a student identified as 2e. Of these, 10 were in the control condition and 14 were in the experimental condition. In the follow-up survey, 51% of teachers (*n* = 27) indicated that they had worked with a student identified as 2e; 14 teachers in the control condition and 13 teachers in the experimental condition. Descriptive statistics for individual items are reported as supplemental data in Table 1.

**Table 1:** Teachers' beliefs about students identified as twice exceptional: item scores.

	**Pre**		**Post**	
	**Experimental**	**Control**	**Experimental**	**Control**
	***M* (*SD*)**	***M* (*SD*)**	***M* (*SD*)**	***M* (*SD*)**
**Beliefs about the characteristics and behaviors of 2e students**				
1. Twice-exceptional students demonstrate advanced reasoning skills.	4.75	4.24	5	4.76
	(0.93)	(1.13)	(0.77)	(0.66)
2. Twice-exceptional students ask complex questions about topics of interest	5.46	4.76	5.5	5.04
	(0.69)	(1.05)	(0.69)	(0.84)
3. Twice-exceptional students show difficulty in written language.	4.36	4.08	4.64	3.96
	(1.22)	(1.38)	(1.03)	(1.17)
4. Twice-exceptional students share complex or advanced information/ideas.	5.18	4.52	5.32	4.96
	(0.72)	(1.16)	(0.86)	(0.68)
5. Twice-exceptional students show potential for expertise in topics of interest.	5.71	5.16	5.68	5.52
	(0.53)	(0.75)	(0.48)	(0.66)
6. Twice-exceptional students demonstrate uneven processing skills (respond slowly, work slowly, or appear to think slowly).	4.57	4.36	4.43	4.28
	(1.37)	(1.19)	(1.37)	(0.89)
7. Twice-exceptional students possess limited social awareness.	4.96	4.32	4.86	4.48
	(0.96)	(1.07)	(0.89)	(1.09)
8. Twice-exceptional students demonstrate uneven academic skills.	4.79	4.56	4.68	4.6
	(1.16)	(1.16)	(1.89)	(0.91)
9. Twice-exceptional students have problems with short-term memory.	3.46	3.44	3.46	3.52
	(1.04)	(1.16)	(1.04)	(1.01)
10. Twice-exceptional students demonstrate unique sense of humor.	4.89	4.48	4.96	4.72
	(0.92)	(0.96)	(1.17)	(1.02)
11. Twice-exceptional students demonstrate sustained engagement at times.	4.46	3.76	4.79	4.28
	(1.07)	(1.23)	(0.99)	(1.17)
12. Twice-exceptional students possess high abilities but may be disruptive in the classroom.	4.96	4.56	4.82	4.72
	(0.96)	(1.16)	(0.94)	(0.98)
**Beliefs about pedagogical programming for 2e students**			
13. Twice-exceptional students will benefit from advanced curricula.	5.18	4.72	5.39	5.16
	(0.98)	(1.10)	(0.79)	(0.63)
14. Twice-exceptional students should participate in accelerated and/or enriched gifted programming.	5.29	4.6	5.25	4.8
	(0.81)	(1.23)	(0.84)	(0.96)
15. Twice-exceptional students' academic needs should be reviewed using flexible screening and identification procedures for involvement in gifted programs.	5.54	4.4	5.36	5.08
	(0.79)	(1.15)	(0.91)	(0.99)
16. Twice-exceptional students will benefit from working with intellectual peers.	5.32	4.84	5.04	5.12
	(0.77)	(0.85)	(1.17)	(1.01)
17. Twice-exceptional students will benefit from strength-based approaches focusing on academic needs.	5.61	4.88	5.25	5.16
	(0.57)	(0.88)	(1.00)	(0.75)
18. Twice-exceptional students will benefit from social and/or emotional support.	5.61	5.36	5.54	5.76
	(0.57)	(0.76)	(0.51)	(0.44)
19. Twice-exceptional students need programming linked to obvious and emergent talents.	5.36	4.56	5.25	5.28
	(0.73)	(1.12)	(0.84)	(0.79)
20. Twice-exceptional students will benefit from gifted programming focused on their talents.	5.61	4.96	5.36	5.16
	(0.57)	(0.98)	(0.78)	(0.69)
21. Twice-exceptional students need access to differentiation approaches honoring high abilities and learning challenges.	5.43	5.04	5.46	5.36
	(0.92)	(0.79)	(0.74)	(0.64)
22. Twice-exceptional students will be successful when teachers are willing to address strengths over learning deficits.	5.64	5	5.54	5.64
	(0.56)	(0.96)	(0.69)	(0.57)

#### Analysis of variance

**Figure 1:**
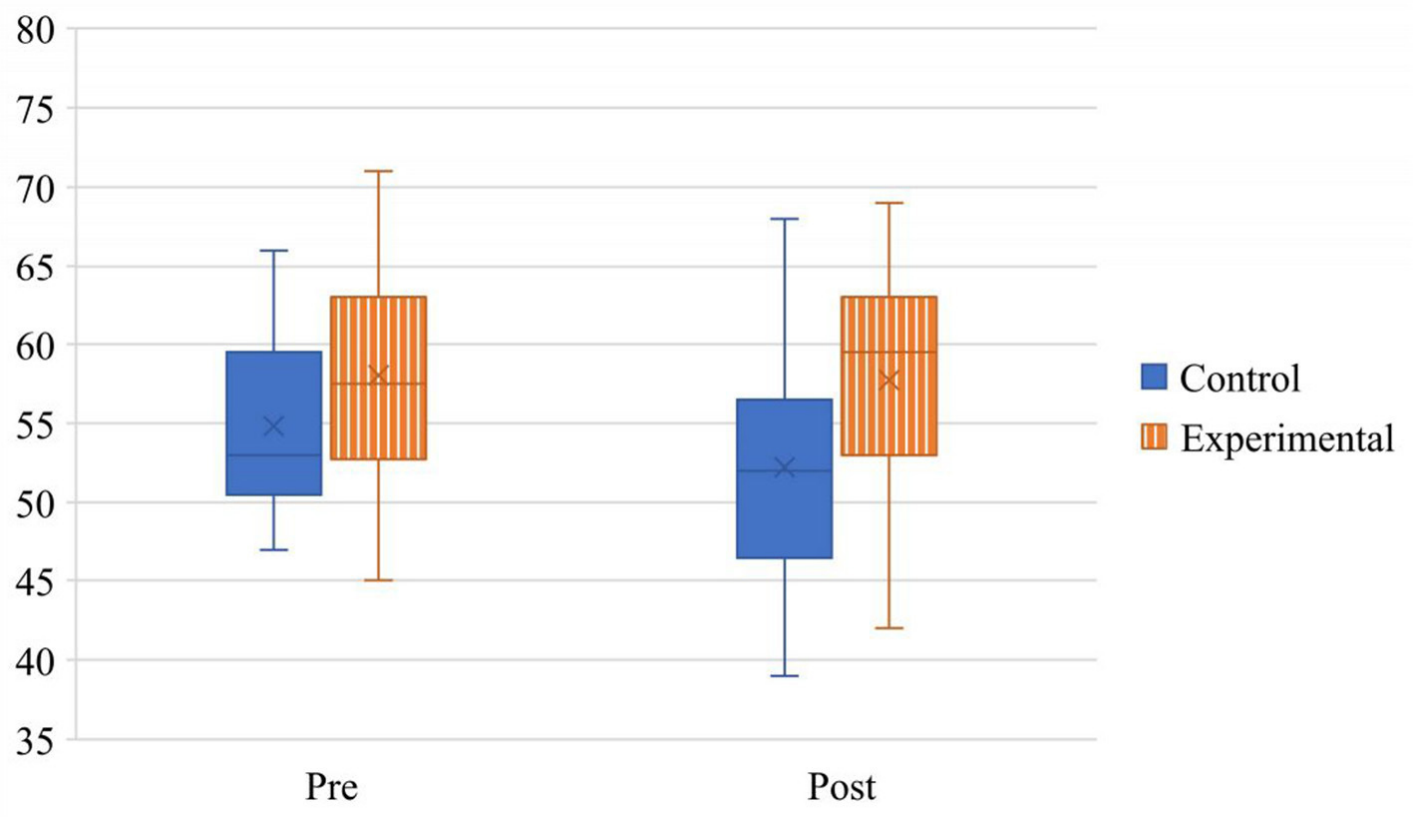
Main effect of condition on teachers' beliefs about the characteristics and behaviors of students identified as 2e.

To address research question (a) and determine whether teachers' reported beliefs about giftedness in students identified as 2e improved after participation in relevant professional learning opportunities, a two-way, mixed ANOVA was used to assess time and condition effects and their interactions for both scales of the Teachers' Beliefs About Twice-Exceptional Students survey. The analysis of teachers' beliefs about the characteristics and behaviors of 2e students revealed a main effect of condition, *F* (1, 51) = 7.44, *p* = 0.009, $\eta_{p}^{2}$ = 0.127, in which experimental group scale scores (*M* = 57.86, *SD* = 6.89) were higher than control group scale scores (*M* = 53.54, *SD* = 6.09). A marginal trend emerged regarding the effect of time, *F* (1, 51) = 3.51, *p* = 0.067, $\eta_{p}^{2}$ = 0.064. The time by condition interaction was not significant, *F* (1, 51) = 1.44, *p* =0.236, $\eta_{p}^{2}$ =0.027. See Figure 1 for a depiction of the observed effects.

**Figure 2:**
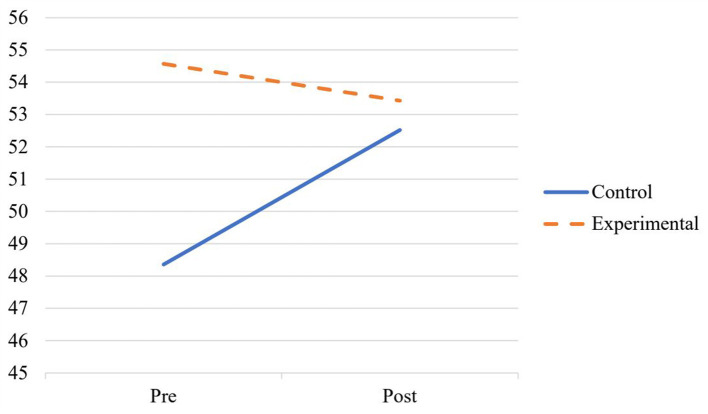
Time by condition interaction on teachers' beliefs about pedagogical programming for students identified as 2e.

The analysis of teachers' beliefs about pedagogical programming for students identified as 2e revealed a main effect of time, *F* (1, 51) = 4.18, *p* = 0.046, $\eta_{p}^{2}$ = 0.076, in which post survey scores (*M* = 53.00, *SD* = 6.27) were higher than pre survey scores (*M* = 51.64, *SD* = 6.08). The main effect of condition, *F* (1, 51) = 6.30, *p* = 0.015, $\eta_{p}^{2}$ = 0.110, indicated that participants in the experimental condition (*M* = 54.00, *SD* = 5.91) scored higher than participants in the control condition (*M* = 50.40, *SD* = 5.98). The time by condition interaction was significant, *F* (1, 51) = 12.91, *p* < 0.001, $\eta_{p}^{2}$ = 0.202; whereas control group scores increased across administrations, scores in the experimental condition slightly decreased (see Figure 2).

### Teachers' reported beliefs about students from CLED populations

#### Descriptive statistics

Collapsed across time periods, teachers reported positive beliefs regarding both the benefits of including students from CLED populations in gifted programs (*M* = 42.21, *SD* = 5.54) and the universality of abilities (*M* = 26.29, *SD* = 2.71), considering the maximum scale scores of 50 and 30, respectively. The scales were strongly correlated (*r* = 0.61, *p* < 0.001). Descriptive statistics for individual items are reported in Table 2.

**Table 2:** Teachers' beliefs about students from CLED populations: item scores.

	**Pre**		**Post**	
	**Experimental**	**Control**	**Experimental**	**Control**
	***M* (*SD*)**	***M* (*SD*)**	***M* (*SD*)**	***M* (*SD*)**
**Benefits of including CLED students in gifted programs**			
1. Broader cultural experiences brought to the gifted classroom by CLED students will benefit students already in gifted programs.	4.32	4.52	4.32	4.48
	(0.72)	(0.59)	(0.67)	(0.66)
2. Gifted students already in gifted programs can benefit from the linguistic abilities of gifted CLED students.	4.25	4.24	4.29	4.36
	(0.70)	(0.78)	(0.66)	(0.86)
3. Modifying instruction to accommodate CLED students does not imply watering down the gifted program.	4.18	4	4.36	4.12
	(0.91)	(0.70)	(0.62)	(0.88)
4. Modifying curriculum to accommodate gifted CLED students will also benefit gifted students already in gifted programs.	3.89	4.12	4.04	4
	(0.79)	(0.83)	(0.79)	(1.04)
5. Attributes of CLED students like resilience and perseverance will benefit students already in gifted programs.	4.14	4.24	4.46	4.56
	(0.65)	(0.66)	(0.70)	(0.65)
6. It is essential that gifted programs include CLED students.	4.18	4.36	4.39	4.56
	(0.77)	(0.64)	(0.83)	(0.58)
7. Gifted CLED students may benefit from a curriculum that recognizes their cultural strength.	4.29	4.2	4.46	4.16
	(0.77)	(0.71)	(0.64)	(0.99)
8. Students already in gifted programs will have fewer services if gifted programs are changed to accommodate CLED students. (Reverse coded)	2.43	2.2	2.14	1.88
	(0.74)	(0.82)	(0.85)	(0.66)
9. Students who have experience in more than one culture have strengths that will help them succeed in gifted programs.	4.07	4	4.25	4.28
	(0.81)	(0.77)	(0.70)	(0.80)
10. The inclusion of CLED students in gifted programs will enhance the multicultural nature of students' learning experience.	4.36	4.24	4.36	4.48
	(0.78)	(0.60)	(0.73)	(0.59)
**Universality of abilities**			
11. Gifted students are found in all economic strata, cultural, and linguistic groups.	4.64	4.52	4.71	4.8
	(0.56)	(0.66)	(0.46)	(0.41)
12. Above average abilities can be demonstrated in many different ways.	4.61	4.48	4.71	4.76
	(0.57)	(0.66)	(0.46)	(0.44)
13. Identification procedures for gifted programs should be constructed in such a way that they identify all students of high ability and potential to achieve.	4.39	4.36	4.68	4.44
	(0.57)	(0.64)	(0.55)	(0.65)
14. CLED students possess the same range of abilities as other students.	4.07	4.28	4.25	4.24
	(0.81)	(0.68)	(0.70)	(0.72)
15. Free or reduced lunch students have a similar range of potential abilities to other students.	4.21	4.16	4.32	4.32
	(0.79)	(0.85)	(0.67)	(0.85)
16. CLED students are able to perform as well as students in advanced academic programs.	3.82	3.92	4.21	4.24
	(0.98)	(0.81)	(0.74)	(0.88)

#### Analysis of variance

To address research question (b) and assess whether teachers' reported beliefs about giftedness in students from CLED populations improved after participation in relevant professional learning opportunities, a two-way, mixed ANOVA was used to analyze time and condition effects and interactions for both scales of the Teachers' Beliefs About Culturally, Linguistically, and Economically Diverse Gifted Students Survey. The two-way, mixed ANOVA assessing teachers' reported beliefs about the benefits of including students from CLED populations in gifted programs revealed a marginal trend regarding administration time, *F* (1, 51) = 3.36, *p* = 0.073, $\eta_{p}^{2}$ = 0.062. Neither the main effect of condition, *F* (1, 51) = 0.95, *p* = 0.759, $\eta_{p}^{2}$ = 0.002, nor the time by condition interaction, *F* (1, 51) = 0.01, *p* = 0.933, $\eta_{p}^{2}$ < 0.001, were significant.

**Figure 3:**
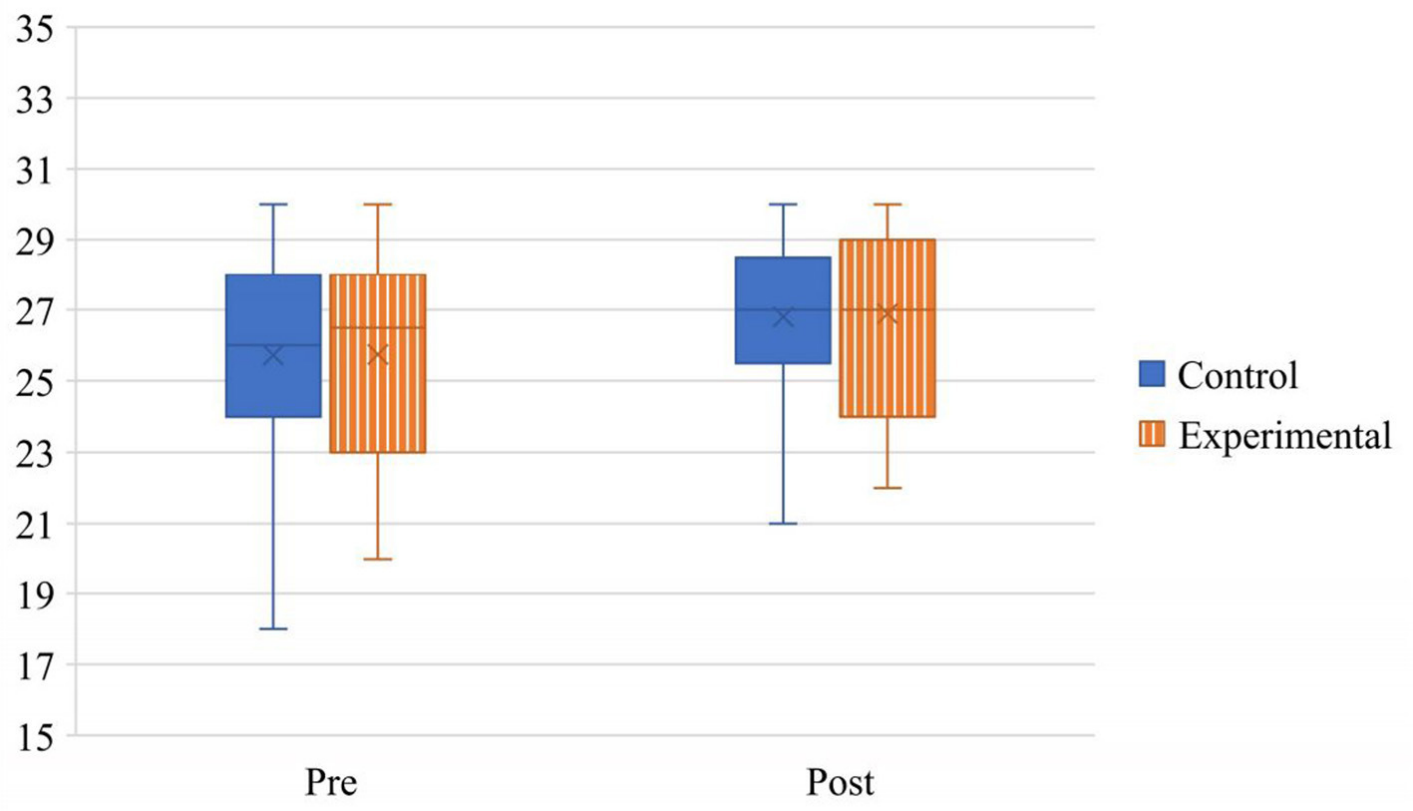
Main effect of time on teachers' beliefs about the universality of abilities.

The analysis of teachers' beliefs about the universality of abilities revealed a main effect of time, *F* (1, 51) = 5.59, *p* = 0.022, $\eta_{p}^{2}$ = 0.099, in which post survey scores (*M* = 26.80, *SD* = 2.54) were higher than pre survey scores (*M* = 25.70, *SD* = 2.78). Neither the main effect of condition, *F* (1, 51) = 0.01, *p* = 0.915, $\eta_{p}^{2}$ < 0.001, nor the time by condition interaction, *F* (1, 51) = 0.00, *p* = 0.947, $\eta_{p}^{2}$ < 0.001, were significant. See Figure 3 for a depiction of the observed effect.

## Discussion

Results related to this study's first research question, which asked whether teachers' reported beliefs about characteristics of giftedness demonstrated by students identified as 2e improved after participation in relevant professional learning opportunities, indicated that there was no significant change in teacher beliefs about this student population.

Similarly, results related to this study's second research question, which asked whether teachers' reported beliefs about giftedness in students from CLED populations improved after participation in relevant professional learning opportunities, indicated that there was no change in teacher beliefs about this student population after experimental teachers' participation in professional learning opportunities.

The findings of this study indicated that teachers from both the experimental and control conditions reported highly accurate views of the characteristics and pedagogical needs of students identified as 2e, and highly positive perceptions of the characteristics of students from CLED populations from the onset of the study. The CLED survey items used in these instruments have previously yielded similarly high initial results (de Wet, 2006; de Wet and Gubbins, 2011), and similar results from the novel 2e survey suggest that teachers hold accurate and positive attitudes toward these populations. As teachers' survey responses were not anonymous, however, it is possible that these results are attributable to high levels of social desirability. Teachers may have also responded to questions defensively because of the pressure they may experience, considering that many educators believe that public perceptions of teachers are overwhelmingly critical (Shine, 2020). Additionally, teachers who chose to participate in this study may have been predisposed to hold certain attitudes involving these student populations prior to their participation, which guided their decision to participate and could have inflated scores.

Results involving teachers' reported beliefs about students identified as 2e indicated that teachers in the experimental condition initially held more positive, accurate beliefs than teachers in the control condition, and both groups' scores marginally increased over time. The main effect of condition confounded the experimental manipulation, as this indicates the two groups varied systematically prior to the intervention. This could be attributable to the fact that, while accommodating for the needs of participating schools, the research team was not able to achieve complete randomization. Unexpectedly, a significant interaction emerged regarding teachers' beliefs about pedagogical programming for students identified as 2e, which indicated that scores increased more in the control group than in the experimental group.

Survey scales assessing teachers' reported beliefs about students from CLED populations demonstrated that teachers' beliefs about both the benefits of including students from CLED populations in gifted programs and the universality of abilities increased over time. The absence of an interaction indicated that this effect did not vary across groups.

As a general result, teachers did not demonstrate a significant change in beliefs due to this study's intervention. These findings suggest that similar professional learning opportunities could fail to produce changes in teacher beliefs over time, which could be attributable to particularly high pre-survey scores or compensatory rivalry. The reported findings support existing research that indicates teacher perceptions are remaining stagnant in relation to the gifted potential among 2e and CLED student populations (Bellara, 2020). Such findings are unexpected, given evidence that students from diverse backgrounds continuously experience disproportionate representation in gifted programs (Hamilton et al., 2018). Given these two seemingly contradictory phenomena, it is important for researchers to carefully consider how they approach studies involving teacher perceptions going forward.

When assessing teacher beliefs, researchers may be unable to determine whether teachers' claims align with their behavior in the classroom. Zheng (2015) argued that teacher beliefs can be understood through the lens of complexity theory, where different agents connect and interact in complex and non-linear systems. He further claimed that the dynamic interactions between different components of these systems complicate the cause-and-effect models that are often assumed when researching teacher assumptions. In some cases, it is possible that perceived norms may guide the way that teachers respond to certain items in survey questionnaires. In addition, teachers might be able to identify or hold positive perceptions of students without transferring these perceptions from understanding to practice. Researchers have reported discrepancies between educator assumptions and educator practices (de Wet, 2006; de Wet and Gubbins, 2011), which poses a validity problem to educational researchers who use teachers' self-reported attitudes to make inferences about subsequent teacher behaviors. Al-Hroub and Whitebread (2008) asserted that teachers' recognition of gifted characteristics in various subpopulations was not ultimately effective if not paired with instructional competence. Even when teachers are motivated to change their practice, they need professional learning opportunities and support to be able to do so, which is not often provided for teachers who wish to expand their understanding of giftedness to include students from diverse cultures (Gubbins et al., 2020).

Theorists exploring the role of teacher beliefs in determining teachers' behavior have claimed that beliefs are instrumental in predicting a person's behavior, which is influenced by an individual's intentions (Borg, 2015; Zheng, 2015). These intentions can be predicted by “attitude, perceived norms, and perceived behavioral control for which beliefs provide the basis” (Karaca and Uysal, 2021, p. 4). Understanding intentions could serve a crucial role in improving instruction, especially when culturally relevant pedagogy is being implemented.

Culturally relevant pedagogy (CRP) is an asset-based model, developed by Ladson-Billings (1995), which emphasizes the importance of student academic success, cultural competence, and critical consciousness. This pedagogy describes using students' cultures as vehicles for learning to enhance their classroom experience. Students', as well as teachers', attitudes, values, and behaviors are strongly influenced by culture. Thus, it is essential that teachers implement culturally responsive practices including: (a) educating students about the diversity of the world around them, (b) promoting equity and mutual respect, and (c) validating students' cultural identity in classroom instructional materials (Krasnoff, 2016). Utilizing CRP strategies is beneficial for all students within the classroom and can help shape teacher beliefs about students from CLED populations. It is important to implement CRP strategies to make students with gifts and talents from CLED populations feel included in the classroom and connected with the content they are learning. This inclusivity is connected to students' academic opportunities as well. A study by Bailey and Rose (2011) indicated that teachers who demonstrated an inclusive philosophy of education were more open to modifying their instructional practices for students of various academic needs.

Implementing CRP strategies with students' cultural strengths in mind has increased student achievement and catalyzed more positive academic self-concepts for students (Long, 2022). Briggs et al. (2008) found that increasing educators' awareness of cultural impact on student academic performance was a key feature to increasing students from CLED populations' participation in gifted programs. Similarly, Ford (2006) defended CRP by explaining that many students want a classroom where “diversity is recognized and honored, and color-blind and culture-blind philosophies are avoided and discouraged” (p. 13). They want teachers to be culturally aware and seek cultural competence. Students also desire access to culturally relevant curricula that is connected to students' backgrounds and personal experiences. Students', as well as teachers', attitudes, values, and behaviors are strongly influenced by culture. Thus, it is essential that teachers implement culturally-responsive practices including: (a) educating students about the diversity of the world around them, (b) promoting equity and mutual respect, and (c) validating students' cultural identity in classroom instructional materials (Krasnoff, 2016). Utilizing CRP strategies is beneficial for all students within the classroom and can help shape teacher beliefs about students from CLED populations.

To optimally support students from CLED populations, it is essential for educators to learn more about CRP and use this knowledge to inform their teaching within the classroom. Garces-Bascal and Elhoweris (2022) emphasized the need for teachers in the field who are committed to CRP and harbor expansive mindsets to begin to counter normative Whiteness and, in turn, make gifted education more equitable. Along with the implementation of CRP attitudes and an increasing of teacher awareness toward culture in the classroom, there must also be changes in behaviors, practices, policies, and procedures to make gifted education more equitable (Ford, 2006; Renzulli and Brandon, 2017; Peters, 2021; Worrell and Dixson, 2021). Teacher beliefs about students in the classroom and subsequent actions are foundational to such changes in the field.

Educational researchers can use the theory of reasoned actions (Fishbein et al., 1980) to emphasize why intentions and teacher beliefs are an important focus for professional learning opportunities (Bianco and Leech, 2010). It follows that “the intentions of effective culturally responsive teachers might better illuminate how they act and to what degree” (Conrad, 2012, p. 91). However, valid measurement of teacher perceptions (and related behaviors) requires that teachers are willing to accurately report their beliefs. To successfully support the academic needs and potential of students identified as 2e and students from CLED populations, educators must couple reported positive perceptions of students from these populations with an observable commitment to creating engaging classroom environments conducive to learning at high levels (Ford and Trotman, 2001). Karaca and Uysal (2021) suggested that beliefs shape intention and intention shapes behavior, but it is possible that the beliefs teachers report do not always shape teachers' intentions or behaviors. Additionally, systemic racism, intensive pressures on teachers, highly homogeneous classrooms, and a lack of time and relevant resources represent significant systemic barriers that are not necessarily overcome by accurate and positive teacher beliefs alone.

This study was informed by a social constructivist perspective on learning and teaching. Previous research involving teacher perceptions of twice-exceptional learners by Bailey and Rose (2011) has indicated the utility of constructivist paradigms to highlight participants' collective generation of meaning. The social constructivist framework views teachers as learners themselves, actively participating in their own learning through the observation and recognition of the experiences and perspectives of others (Harkness, 2009; Armstrong, 2019). Framing studies through the social constructivist view can help initiate the reconceptualization of teacher knowledge and teacher beliefs from subjective understanding to inter-subjective understanding (Nagamine, 2007). Teachers work together to interpret new information related to their educational practices. Additionally, teachers may work alongside parents to co-construct an understanding of students identified as 2e (Mollenkopf et al., 2021) as well as students from CLED populations.

Understanding that previously constructed knowledge is continuously modified and tested through shared, as well as individual, experiences is imperative when promoting teachers' application of newly acquired knowledge. This reframing may elevate researchers' understanding of how socially constructed learning takes form among educators as well as how this can be leveraged to benefit students from diverse backgrounds who have historically been underrepresented in gifted education programs. The TLM math unit supported the growth of educators through its integration as an educative curriculum. Therefore, while students were learning the material in the unit, teachers were simultaneously gaining pedagogical content knowledge and skills regarding implementing the material (Davis and Krajcik, 2005). The unit supplied teachers with an opportunity to reflect upon their role and actions within the classroom, similar to how the teachers reflected on their beliefs regarding students from 2e and CLED backgrounds while completing the surveys. Further, the professional learning allowed teachers to hold a holistic perspective and examine research within the field of gifted education. An external outlook allowed teachers to examine disproportionalities for diverse students in gifted education, teachers' beliefs about gifted students from diverse backgrounds, and, thus, how these beliefs impact students in classrooms and gifted programs.

Because this study reflects highly positive and accurate teacher beliefs about students among 2e and CLED populations, but these same students are disproportionately under-identified for gifted education services (Hamilton et al., 2018), further research is needed to better comprehend the implications of teachers' socially constructed beliefs about these populations (Foley-Nicpon et al., 2011, 2015). A recent study (Dimitriadis et al., 2021) revealed that teachers who received professional learning opportunities in identifying the needs of students identified as 2e did not demonstrate increased knowledge and confidence about how to instruct this population of students, when compared to teachers who did not receive this professional learning. These findings suggest that professional learning may need to include a more specific focus on teacher practices, rather than teacher perceptions, to help support the needs of students from historically underserved communities. The National Center for Research on Gifted Education (n.d.) provided tips for improving the identification of ELs, such as the adoption of universal screening processes, the creation of alternative pathways to identification, and the establishment of communication systems that may result in positive academic outcomes for students from CLED populations. There has also been a call for increased attention and major societal changes to address equity-related issues (Lamb et al., 2021; Makel, 2021; McCoach, 2021). Walrod (2022) discussed changes in protective laws (e.g., IDEA) as well as an increase in effective professional learning for relevant stakeholders. The present research recommends that future studies and professional learning efforts involving teacher perceptions be used to connect teacher perceptions with their behaviors in the classroom. In such research, more sensitive measures and interventions may be needed to determine how to best change teachers' attitudes and corresponding behaviors.

## Limitations

The limitations of study implementation are based on the time commitment and the goal of random assignment. First, the professional learning was 2 days for teachers in the experimental condition. This timeframe may have been too short for an accurate reflection on teachers' reported beliefs when they are introduced to identifying and serving students with gifts in talents who have been historically underrepresented in gifted and talented programs.

Second, researchers in this study were not able to fully randomize group assignment, which may have resulted in systematic differences between teachers in control and experimental groups. School administrators who were aware of the nature of the study may have led researchers to teachers in both conditions who already demonstrated highly accurate, or positive, views of students among 2e and CLED populations, resulting in artificially high pre-implementation questionnaire scores. Teachers in the control group were not blinded to the condition assignment and were likely in contact with the teachers who were in the experimental condition. Researchers were not aware of the extent to which teachers in both conditions were able to converse with each other about their experiences with the study. This may have led to treatment diffusion and reduced the treatment's effect. Furthermore, this study only included measures of self-reported attitudes. These attitudes were not observed alongside classroom behavior, precluding any inferences about effects within classrooms. Without additional measures, the study could not account for the compensatory rivalry that may have inspired teachers in the control group to report specific beliefs about students identified as 2e and students from CLED populations within gifted education programs.

## Data availability statement

The original contributions presented in the study are included in the article/supplementary material, further inquiries can be directed to the corresponding author/s.

## Ethics statement

The studies involving human participants were reviewed and approved by Institutional Review Board. The patients/participants provided their written informed consent to participate in this study.

## Author contributions

EG designed and supervised the study, developed and piloted the surveys, collected data, validated the results, drafted, reviewed, and revised the manuscript. RC and EC drafted, reviewed, and revised the manuscript. GB performed the data analysis and drafted, reviewed, and revised the manuscript. SH organized participant's recruitment, collected data, and proofread the manuscript. AB developed and piloted the surveys, performed the data analysis, and proofread the manuscript. KK proofread the manuscript. All authors contributed to the article and approved the submitted version.

## Funding

This work was supported by Jacob K. Javits Gifted and Talented Students Education Program, United States Department of Education PR/Award # S206A170023, Thinking Like Mathematicians: Challenging All Grade 3 Students.

## Conflict of interest

The authors declare that the research was conducted in the absence of any commercial or financial relationships that could be construed as a potential conflict of interest.

## Publisher's note

All claims expressed in this article are solely those of the authors and do not necessarily represent those of their affiliated organizations, or those of the publisher, the editors and the reviewers. Any product that may be evaluated in this article, or claim that may be made by its manufacturer, is not guaranteed or endorsed by the publisher.
